# Isometric contraction induces transient increase of REDD1 expression in non‐contracted muscles partly through glucocorticoids

**DOI:** 10.14814/phy2.15745

**Published:** 2023-06-06

**Authors:** Taro Murakami

**Affiliations:** ^1^ Department of Nutrition Shigakkan University Obu Japan

**Keywords:** isometric contraction, mTORC1, muscle protein synthesis, REDD1, RU‐486

## Abstract

This study investigated whether muscle contraction induces expression of regulated in development and DNA damage 1 (REDD1), a potent inhibitor of mTORC1, in mice muscle. Gastrocnemius muscle was unilaterally and isometrically contracted with electrical stimulation, and changes in muscle protein synthesis, mTORC1 signaling phosphorylation, and REDD1 protein, and mRNA were measured at time points of 0, 3, 6, 12, and 24 h after the contraction. At time point 0 and 3 h, muscle protein synthesis was blunted by the contraction, accompanied by a decrease in phosphorylation of 4E‐BP1 at time point 0 h, suggesting suppression of mTORC1 was involved in blunting of muscle protein synthesis during and shortly after the contraction. REDD1 protein was not increased in the contracted muscle at these time points, but at time point 3 h, both REDD1 protein and mRNA were increased in the contralateral non‐contracted muscle. The induction of REDD1 expression in the non‐contracted muscle was attenuated by RU‐486, an antagonist of the glucocorticoid receptor, suggesting that glucocorticoids are involved in this process. These findings suggest that muscle contraction induces temporal anabolic resistance in non‐contracted muscle, potentially increasing the availability of amino acids for contracted muscle, allowing for the synthesis of muscle protein.

## 1 INTRODUCTION

Organisms strictly but flexibly control a hierarchy of adenosine triphosphate (ATP)‐consuming processes to maintain energy homeostasis (Buttgereit & Brand, 1995). During exercise, ATP expenditure for muscle contraction takes priority over synthesizing macromolecules such as proteins (Bylund‐Fellenius et al., 1984). One of the mechanisms by which muscle contraction can suppress protein synthesis is by decreasing the activity of mammalian target of rapamycin (mTOR) complex 1 (mTORC1; Gautsch et al., 1998), which regulates key cellular functions linked to the promotion of cell growth and metabolism (Kimball & Jefferson, 2010; Liu & Sabatini, 2020; Shimobayashi & Hall, 2014). Adenosine monophosphate (AMP)‐activated protein kinase (AMPK; Dreyer et al., 2006; Williamson et al., 2006) and regulated in development and DNA damage response 1 (REDD1; Murakami et al., 2011) are proposed candidates that drive exercise‐induced decrease in mTORC1 signaling. In addition, Ca^2+^‐dependent inactivation of eukaryotic elongation factor 2 (eEF2), which regulates the elongation step in protein synthesis, has also been reported to be involved in suppressing protein synthesis in working muscles (Rose et al., 2005, 2009).

REDD1 is transcriptionally upregulated in response to various stress conditions, including ATP depletion (Sofer et al., 2005), DNA damage (Lin et al., 2005), endoplasmic reticulum stress (Protiva et al., 2008; Wang et al., 2003), hypoxia (Brugarolas et al., 2004; DeYoung et al., 2008; Favier et al., 2010; Shoshani et al., 2002), glucocorticoid treatment (Wang et al., 2003, 2006), and starvation (McGhee et al., 2009). It inhibits mTORC1 by activating TSC1/TSC2 (Brugarolas et al., 2004; DeYoung et al., 2008; Sofer et al., 2005), and it has been shown to be a transient inhibitor of mTORC1 during exercise (Gordon et al., 2017; Hayasaka et al., 2014; Murakami et al., 2011), with a short half‐life of less than 5 min (Kimball et al., 2008).

Endurance exercise has been shown to increase the REDD1 mRNA and protein contents and decrease mTORC1 in rat skeletal muscle (Gordon et al., 2017; Hayasaka et al., 2014; Murakami et al., 2011), while resistance exercise has been reported to decrease REDD1 mRNA in human muscle (Drummond et al., 2008; Liu et al., 2010). Moreover, an electrical stimulation‐evoked eccentric contraction decreases REDD1 protein content in association with an increase in mTORC1 and protein synthesis in mice muscle (Gordon et al., 2014). Although electrical stimulation‐evoked eccentric and/or isometric contraction could not perfectly replicate physiologic resistance exercise, they are informative models to elucidate the mechanisms by which contraction increases muscle protein synthesis (Ashida et al., 2018; Baar & Esser, 1999; Gordon et al., 2014; Maruyama et al., 2020). One reason for the discrepancy in REDD1 expression between endurance exercise and resistance exercise or eccentric contraction may be the time point at which REDD1 is measured after the exercise. REDD1 expression has been measured immediately after endurance exercise (Gordon et al., 2017; Hayasaka et al., 2014; Murakami et al., 2011) and 4 h after resistance exercise (Drummond et al., 2008; Liu et al., 2010), as well as 0.5 and 4 h after eccentric contraction (Gordon et al., 2014). Since both types of exercise and eccentric contraction require ATP for contraction and disrupt energy homeostasis in the muscle, suggesting that they may increase REDD1 expression during exercise and eccentric contraction.

In this study, the aim was to investigate whether isometric contraction increases REDD1 expression immediately after the contraction. The results showed that unilateral isometric contraction of the mice gastrocnemius muscle did not increase REDD1 protein immediately after the contraction. However, isometric contraction increased REDD1 protein and mRNA in the contralateral non‐contracted muscle 3 h after the contraction. Moreover, the isometric contraction‐induced increases in REDD1 protein and mRNA were completely attenuated in the contracted muscle.

## 2 MATERIALS AND METHODS

### 2.1 Animals

Male C57BL/6J mice (9 weeks old) were obtained from Japan SLC Inc. (Hamamatsu, Japan). The Experimental Animal Care Committee of Shigakkan University approved all procedures involving animals. Mice were housed at 22°C and 50% humidity, with a 12:12 h light–dark (12 L/12D) photoperiod. Food (CE‐2; CLEA Japan) and water were provided ad libitum. All experiments were carried out after 1 week of acclimatization to the environment and mice were reached to 10 weeks old.

### 2.2 Muscle contraction protocol

The gastrocnemius muscle was unilaterally isometric contracted as described by Maruyama et al (2020). Briefly, under isoflurane anesthesia (2%–3% inhalation), the right gastrocnemius muscle was contracted isometrically via percutaneous electrical stimulation (100 Hz, five sets of ten 3‐s contractions, 7‐s rest between contractions, 3‐min rest between sets). The voltage (30 V) and stimulation frequency were adjusted to produce maximum isometric tension (Ashida et al., 2018; Zhao et al., 2017). The left gastrocnemius muscle served as a control. Muscle samples were obtained 0, 3, 6, 12 and 24 h after the contraction (*n* = 6). To determine the rate of muscle protein synthesis, anesthetized mice (2% isoflurane inhalation) at each time point were administered puromycin (cat. no. P8833, Sigma‐Aldrich) intraperitoneally (40 μmol/kg of body weight) and euthanized by cervical dislocation exactly 15 min later while still under anesthesia. The gastrocnemius muscle was quickly removed and frozen in liquid nitrogen. Samples were stored at −80°C until use. In the experiment for comparing the REDD1expression from contracted and non‐contracted animals, a subset of mice was prepared. Gastrocnemius and triceps brachii muscle samples from both animals (*n* = 5) were obtained 3 h after the contraction of contracted animals as described above without puromycin administration.

### 2.3 RU‐486 treatment

As reported in previous studies, muscle REDD1 expression can be induced by glucocorticoids, such as dexamethasone (Wang et al., 2003, 2006). To explore the role of glucocorticoids in REDD1 expression, a subset of mice (*n* = 6) was administered RU‐486, an effective antiglucocorticoid that has shown clinical efficacy in function (Cadepond et al., 1997). RU‐486 (cat. no. 10006317, Cayman Chemical) diluted in polyethylene glycol 400 (PEG 400; cat. no. 161–09065, Fiji Film Wako) was administered subcutaneously (40 mg/kg body weight) to mice 16 and 2 h prior to the start of the isometric contraction protocol, as described by Kumari et al (2011). The control group (*n* = 6) received two injections of PEG 400. To examine whether isometric contraction increases serum corticosterone concentration, another control group of mice (*n* = 6), which were not subjected to the contraction, was prepared. These mice also received two injections of PEG 400. Blood was collected from the inferior vena cava of all mice groups under isoflurane (2% inhalation) anesthesia 3 h after the contraction. The mice were then euthanized by cervical dislocation while still under anesthesia, and gastrocnemius muscle was collected as described above. The collected blood was allowed to clot on ice for ≥30 min, followed by centrifugation (2,000 g for 10 min at 4°C). The obtained serum was stored at −80°C for further analysis.

### 2.4 Serum corticosterone

Serum corticosterone levels were determined by ELISA (#K014‐H1, Arbor Assays) according to the manufacturer's procedures.

### 2.5 Western blot

Western blot including preparation of muscle extract was carried out as described previously (Murakami et al., 2011). In brief, protein extracts from gastrocnemius muscle (25 μg) were separated using 10% sodium dodecyl sulfate polyacrylamide gel electrophoresis (SDS‐PAGE). The resolved samples were transferred onto 0.45 μm polyvinylidene fluoride (PVDF) membranes (Millipore, Billerica, MA) using a semi‐dry blotter (Bio‐Rad Laboratories) and blocked for 1 h with PVDF Blocking Reagent of Can Get Signal (Toyobo). The membranes were then incubated overnight at 4°C with the following antibodies: phospho‐T389 S6K1 (#9234), Total S6K1 (#2708), phospho‐T37/T46 4E‐BP1 (#2855), total 4E‐BP1 (#9644), phospho‐T172 AMPKα (#2535), and total AMPKα (#5831) obtained from Cell Signaling Technology; REDD1 (#10638‐1‐AP) obtained from Proteintech Group; and β‐actin (#sc‐81,178) obtained from Santa Cruz Biotechnology. All primary antibodies were diluted 4,000 times in a solution of Can Get Signal Solution 1 (Toyobo). After washing the membranes with 0.1% Tween 20 containing Tris‐buffered saline (TBST), the membranes were incubated with the secondary antibody (Bio‐Rad Laboratories, Inc.) at room temperature for 1 h in a solution of 3% skim milk in TBST (for β‐actin) or Can Get Signal Solution 2 (for antibodies other than β‐actin, Toyobo). The secondary antibody was diluted 10,000 times in the solutions. Blots were developed using enhanced chemiluminescence prime western blotting detection reagents (GE Healthcare UK). The bands were scanned with Image Quant LAS500 (GE Healthcare UK), and the intensities were analyzed using ImageJ software. The intensity of the band of the phosphorylated form of 4E‐BP1 or T389 was divided by that of the total form and expressed as a relative value to the mean value of non‐contracted muscles at the time point 0 h. The intensity of the band of REDD1 in the time course experiment was divided by that of Coomassie brilliant blue (CBB)‐stained whole‐lane protein and expressed as a relative value to the mean value of non‐contracted muscles at the time point 0 h. The intensity of REDD1 and phosphorylated AMPK bands in the time course experiment were normalized to that of Coomassie brilliant blue (CBB)‐stained whole‐lane protein, as the β‐actin protein band was affected by the contraction. The data were expressed as relative values to the mean value of non‐contracted muscles at time point 0 h. For experiments other than the time course experiment, the intensity of REDD1 protein bands was normalized to that of β‐actin and expressed as a relative value to the mean value of non‐contracted muscles (left limbs) of contracted animals.

### 2.6 Protein synthesis

The rate of protein synthesis was measured by the SUnSET method (Schmidt et al., 2009) using an anti‐puromycin monoclonal antibody (#MABE343, Merck Millipore) as described previously (Hayasaka et al., 2014).

### 2.7 qRT‐PCR

REDD1 mRNA was measured with qRT‐PCR as described previously (Murakami et al., 2011). The primers sequences were as follows: 5’‐TGGTGCCCACCTTTCAGTTG‐3′ (forward), 5’‐GTCAGGGACTGGCTGTAACC‐3′ (reverse) for REDD1 (Kimball et al., 2008) and 5’‐CGAACGTCTGCCCTATCAAC‐3′ (forward), 5’‐GCCTTCCTTGGATGTGGTAG‐3′ (reverse) for 18S rRNA (Murakami et al., 2011). The relative standard curve method was used to analyze the data using 5 points of the standard and expression level of REDD1 mRNA was normalized to that of 18S rRNA.

### 2.8 Statistical analysis

To evaluate the differences between the groups, most data were analyzed using a two‐way repeated measures analysis of the variance (ANOVA). A one‐way ANOVA was used for analyzing serum corticosterone concentration. A paired Student's *t*‐test was used for analyzing REDD1 mRNA at 3 h after the contraction. A Tukey–Kramer test (for comparison among the non‐contracted or the isometrically contracted muscles at different time points) or a paired Student's *t*‐test (for comparison between the non‐contracted and the isometrically contracted muscles at the same time point) were used for the post hoc test when a significant difference was observed in ANOVA. Values of *p* < 0.05 were defined as statistically significant.

## 3 RESULTS

Incorporation of puromycin into newly synthesized peptides were transiently decreased by isometric contraction.

**FIGURE 1:**
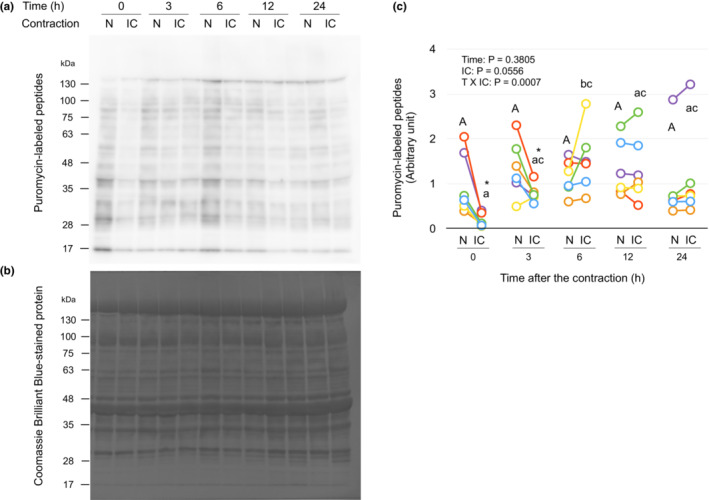
Transient decrease in puromycin incorporation into newly synthesized peptides following isometric contraction. The right gastrocnemius muscle of mice was isometrically contracted as described in “Materials and Methods” and both right and left gastrocnemius muscles were collected over 24 h after the contraction. The contralateral (left) non‐contracted muscle was provided as a control. The incorporation of puromycin into newly synthesized peptides was measured by administration of puromycin into mice followed by immunoblot with anti‐puromycin antibody (SUnSET method; Kumari et al., 2011). (a) Representative immunoblots. (b) Coomassie brilliant blue (CBB)‐stained whole‐lane protein. (c) Changes in puromycin‐labeled peptides by isometric contraction. The intensity of bands of puromycin‐labeled peptides was divided by that of CBB‐stained whole‐lane protein and expressed as the relative value to the mean value of non‐contracted muscles at time point 0 h. Each connected circle shows the data obtained from the same animal (*n* = 6 for each time point). Values of means ± SD are listed in Table S1 (https://figshare.com/s/7910d6fd80e7261298f4). Data not sharing the same capital letter (among the non‐contracted muscles) or small letter (among the isometrically contracted muscles) are significantly different between time points after the contraction (*p* < 0.05). An asterisk (*) indicates significant differences between non‐contracted muscles and isometrically contracted muscles at the same time point (*p* < 0.05). N, non‐contracted muscle; IC, isometrically contracted muscle.

The incorporation of puromycin into newly synthesized peptides were significantly decreased in the contracted muscle at time point 0 and 3 h, after that the incorporation were not different between contracted and contralateral non‐contracted muscle at time points 6, 12 and 24 h (Figure 1). In a pilot study, it was confirmed that the concentration of free puromycin in the muscle was not different between contracted and non‐contracted muscles after the contraction at time point 0 h, suggesting that puromycin was delivered equally to both muscles (Figure S1 (https://figshare.com/s/7910d6fd80e7261298f4)). These results suggest that muscle protein synthesis is blunted during and shortly after the isometric contraction.

Phosphorylation of mTORC1 signaling is transiently decreased by isometric contraction.

**FIGURE 2:**
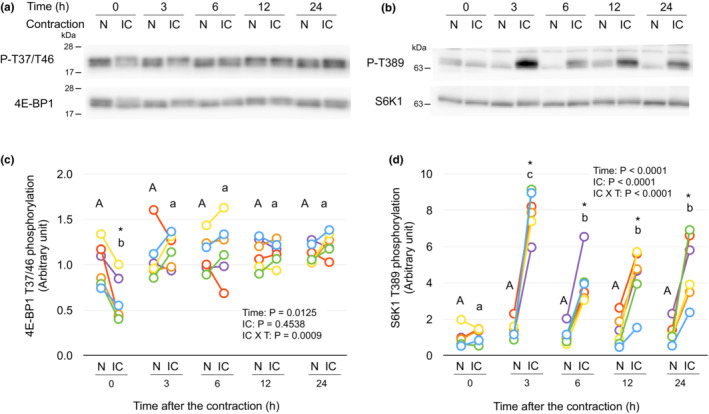
Phosphorylation of mTORC1 signaling is transiently decreased by isometric contraction. (a,b) Representative immunoblots of phosphorylation of 4E‐BP1 (T37/T46) and S6K1 (T389), respectively. (c,d) Changes in phosphorylation of 4E‐BP1 and S6K1, respectively, by the isometric contraction. The intensity of band of phosphorylated form (T37/T46 or T389) was divided by that of total form (4E‐BP1 or S6K1) and expressed as the relative value to the mean value of non‐contracted muscles at time point 0 h. Each connected circle shows the data obtained from same animal (*n* = 6 for each time point). Values of means ± SD are listed in Table S1 (https://figshare.com/s/7910d6fd80e7261298f4). Data not sharing the same capital letter (among the non‐contracted muscles) or small letter (among the isometrically contracted muscles) are significantly different between time points after the contraction (*p* < 0.05). An asterisk (*) indicates significant differences between non‐contracted muscles and isometrically contracted muscles at the same time point (*p* < 0.05). N, non‐contracted muscle; IC, isometrically contracted muscle.

Phosphorylation of T37/T46 of 4E‐BP1 was significantly decreased in the contracted muscle at time point 0 h, after that the phosphorylation was not different between both muscles over 24 h (Figure 2a,c). Phosphorylation of T389 of S6K1 was not different between contracted and contralateral non‐contracted muscle at time point 0 h, and the phosphorylation was increased in the contracted muscle after 3 h of the contraction over 24 h (Figure 2b,d). These results suggest that mTORC1 signaling is blunted during isometric contraction. The blunted mTORC1 signaling may contribute to the decrease in protein synthesis during and shortly after the contraction.

REDD1 protein and mRNA are increased in non‐contracted muscle, but not in contracted muscle by isometric contraction.

**FIGURE 3:**
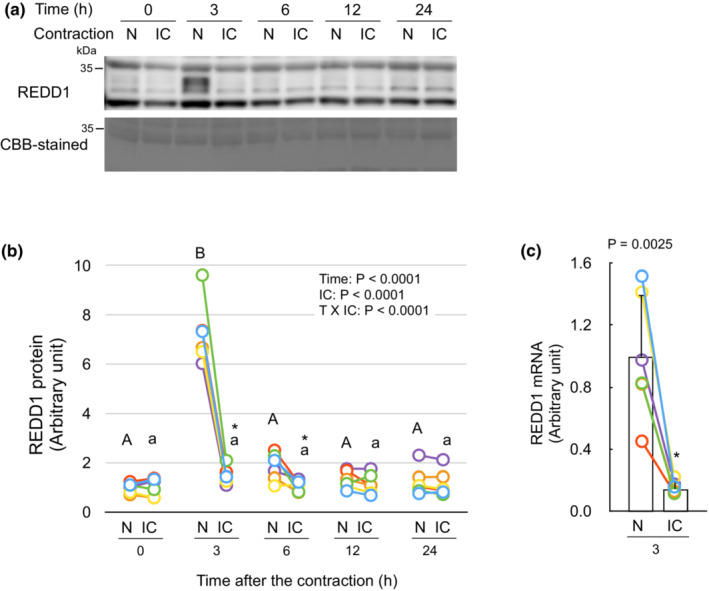
REDD1expression is increased in non‐contracted muscle, but not in contracted muscle by isometric contraction. (a) Representative immunoblots of REDD1 (upper panel) and Coomassie brilliant blue (CBB)‐stained protein of an applicable area (lower panel). (b) Changes in REDD1 protein by the isometric contraction. The intensity of band of REDD1 protein was divided by that of CBB‐stained whole‐lane protein and expressed as the relative value to the mean value of non‐contracted muscles at time point 0 h. (c) The mRNA of REDD1 in the muscle at time point 3 h was measured with qRT‐PCR. Each connected circle shows the data obtained from same animal (*n* = 6 for each time point). Values of means ± SD for REDD1 protein are listed in Table S1 (https://figshare.com/s/7910d6fd80e7261298f4). Data not sharing the same capital letter (among the non‐contracted muscles) or small letter (among the isometrically contracted muscles) are significantly different between time points after the contraction (*p* < 0.05). An asterisk (*) indicates significant differences between non‐contracted muscles and isometrically contracted muscles at the same time point (*p* < 0.05). N, non‐contracted muscle; IC, isometrically contracted muscle.

**FIGURE 4:**
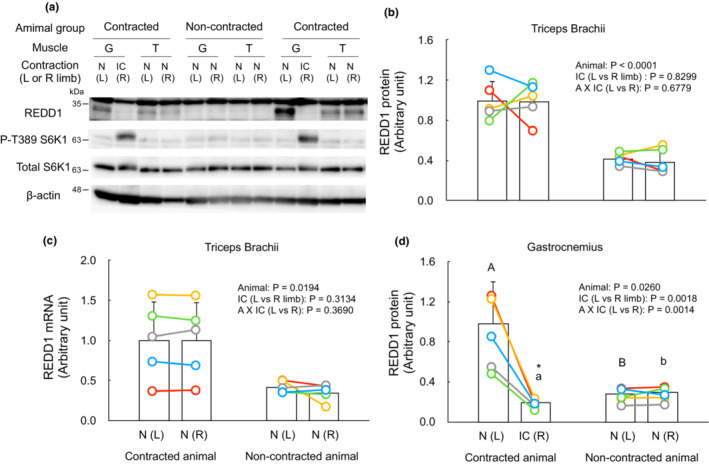
Isometric contraction of gastrocnemius muscle induces REDD1 expression in triceps brachii muscles. Mice were divided into contracted and non‐contracted animal groups (*n* = 5 for each animal group). The right gastrocnemius muscle of mice of the contracted animal group was isometrically contracted as described in “Materials and Methods”. Three hours after the contraction, the gastrocnemius and triceps brachii muscles of mice of both animal groups were collected. The proteins of REDD1, phospho‐ and total S6K1, and β‐Actin were measured with immunoblot. The mRNA of REDD1 in the triceps brachii muscle was measured with qRT‐PCR. (a) Representative immunoblots. (b,c) The REDD1 protein and mRNA of brachii muscles, respectively. (d) The REDD1 protein of the gastrocnemius muscle. The intensity of bands of the REDD1 protein was divided by that of β‐Actin. The values are expressed as the relative value to the mean value of non‐contracted muscles (left limb muscles) from animals of the contracted animal group. Each connected circle shows the data obtained from the same animal. Data with different capital letters (between left limb muscles of different animal groups) or small letters (between right limb muscles of different animal groups) are significantly different (*p* < 0.05). An asterisk (*) indicates significant differences between left and right limb muscles within the same animal group (*p* < 0.05). N, non‐contracted muscle; IC, isometrically contracted muscle. L, left limb muscle; R, right limb muscle.

At time point 3 h, REDD1 protein was found to be increased only in the contralateral non‐contracted muscle (Figure 3a,b), along with an upregulation of REDD1 mRNA in the muscle (Figure 3c). At time point 6 h, while the value was not significantly different from other time points, REDD1 protein expression remained increased in the non‐contracted muscle. These results suggest that isometric contraction induces the expression of REDD1 in tissues including non‐contracted muscles. Furthermore, the contraction itself attenuates the contraction‐induced REDD1 expression in the contracted muscle. Indeed, at time point 3 h of isometric contraction of gastrocnemius muscle, REDD1 protein and mRNA were increased in both left and right triceps brachii muscles (Figure 4a–c). However, the REDD1 protein content in the contracted gastrocnemius muscle was lower than that in the muscle from non‐contracted animals (Figure 4d). REDD1 proteins in liver, kidney, and heart were not different between non‐contracted and isometrically contracted mice (Figure S2 (https://figshare.com/s/7910d6fd80e7261298f4)). These results indicate that isometric contraction leads to REDD1 expression in non‐contracted muscles, but not in the contracted muscle.

Inhibition of glucocorticoid receptor suppresses isometric contraction‐induced REDD1 expression in non‐contracted muscle.

**FIGURE 5:**
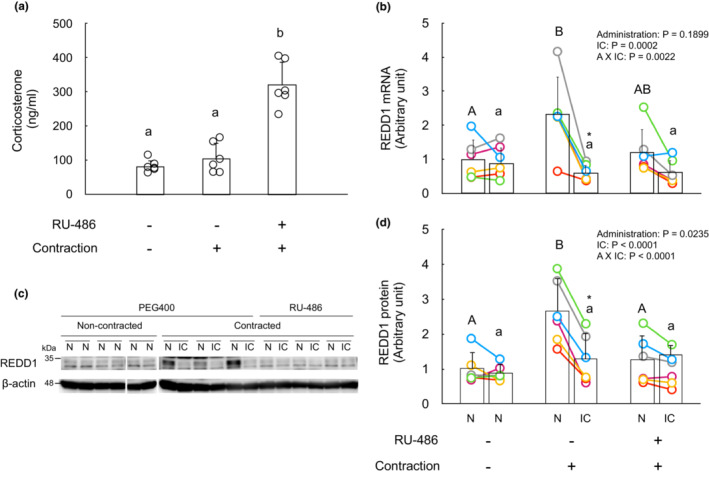
Inhibition of glucocorticoid receptor suppresses isometric contraction‐induced REDD1 expression in non‐contracted muscle. To clarify the involvement of glucocorticoid for REDD1 expression, animals (*n* = 6) were administered RU‐486 diluted in polyethylene glycol 400 subcutaneously (40 mg/kg body weight) at 16 and 2 h prior to the start of isometric contraction protocol. Mice in the control group (*n* = 6) received two injections with solvent (PEG 400). To examine whether the isometric contraction increases serum corticosterone concentration, another control group of mice (*n* = 6), which were not subjected to the contraction was prepared. These mice also received two injections with solvent (PEG400). Three hours after the contraction, blood and gastrocnemius muscle of mice in all groups were collected. (a) Serum corticosterone concentration was measured with ELISA. The REDD1 mRNA (b) and protein (c,d) were measured with qRT‐PCR and Western blot, respectively. White spaces between the bands of Immunoblots (c) indicate that membranes are discontinuous. The intensity of the bands on the different membranes was standardized using internal control bands on each membrane. The intensity of the bands of REDD1 protein was divided by that of β‐Actin and is expressed as the relative value to the mean value of the left limb muscles from control animals not subjected to the contraction. In panel (a), data not sharing the same superscript are significantly different (*p* < 0.05). In panels (b) and (d), data with different capital letters (between the non‐contracted (left limb) muscles of different groups) or small letters (between isometrically contracted (right limb) muscles of different groups) are significantly different (*p* < 0.05). An asterisk (*) indicates significant differences between non‐contracted and isometrically contracted muscles within the same group (*p* < 0.05). N, non‐contracted muscle; IC, isometrically contracted muscle.

Glucocorticoids are known to induce REDD1 expression (Wang et al., 2003, 2006), and resistance exercise is known to transiently increase serum glucocorticoids (Kraemer & Ratamess, 2005). To clarify the involvement of glucocorticoids in REDD1 expression in non‐contracted muscle, I administered RU‐486 (Cadepond et al., 1997), an antagonist of glucocorticoid receptor, to mice before the contraction and determined whether the increase in REDD1 protein and mRNA were attenuated in gastrocnemius muscle. Serum corticosterone concentration was not different between non‐contracted control and contracted animals 3 h after the contraction (Figure 5a). It is noteworthy that RU‐486 increased serum corticosterone concentration in contracted animals (Figure 5a), as observed by Gordon et al (Gordon et al., 2017), suggesting that RU‐486 inhibits the glucocorticoid receptor, and the feedback of ACTH increases the release of corticosterone from adrenal glands. RU‐486 decreased REDD1 protein and mRNA only in non‐contracted but not contracted muscle. These results suggest that although an increase in corticosterone concentration is not observed 3 h after the contraction, a contraction‐induced increase in corticosterone increases REDD1 expression in non‐contracted muscles.

Phosphorylation of AMPK is transiently increased by isometric contraction.

**FIGURE 6:**
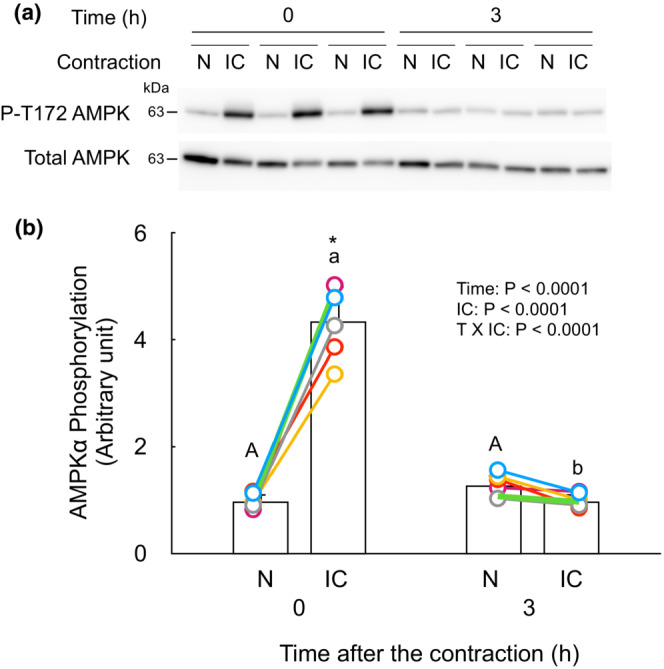
Phosphorylation of AMPK is transiently increased by isometric contraction. No change of REDD1 protein in the contracted muscle at time point 0 h suggest that REDD1 is not involved in the blunting of protein synthesis and mTORC1 signaling. Thus, the phosphorylation/activation of another candidate for blunting mTORC1 signaling, phosphorylation of AMPK was measured using gastrocnemius muscle at time point 0 and 3 h. (a) Representative immunoblots of AMPK. (b) Change in phosphorylation of α subunit of AMPK by the isometric contraction. The intensity of band of phosphorylated form (T172‐AMPK) was divided by that of Coomassie brilliant blue‐stained whole‐lane protein and expressed as the relative value to the mean value of non‐contracted muscles at time point 0 h. Each connected circle shows the data obtained from same animal (*n* = 6 for each time point). Data not sharing the same capital letter (among the non‐contracted muscles) or small letter (among the isometrically contracted muscles) are significantly different between time points after the contraction (*p* < 0.05). An asterisk (*) indicates significant differences between non‐contracted muscles and isometrically contracted muscles at the same time point (*p* < 0.05). N, non‐contracted muscle; IC, isometrically contracted muscle.

No change of REDD1 protein in the contracted muscle at time point 0 h suggests that REDD1 is not involved in the blunting of protein synthesis and mTORC1 signaling. Phosphorylation of T172 of α subunit of AMPK was increased in the contracted muscle at time point 0 h but not at time point 3 h (Figure 6). The activation of AMPK is likely involved in the blunting of protein synthesis and mTORC1 signaling during and shortly after the isometric contraction.

## 4 DISCUSSION

The aim of this study was to determine whether REDD1 expression is increased in isometrically contracting muscles. Contrary to expectations, the REDD1 protein was not increased in the contracted gastrocnemius muscle just after the contraction. However, the REDD1 protein and mRNA were found to be increased in contralateral non‐contracted muscle 3 h after the contraction. This increase was also observed in both left and right triceps brachii muscles by the contraction of gastrocnemius muscle. It should be noted that the mice used in this study were relatively young (10 weeks old), which may have influenced the observed results due to the period of rapid growth. However, in my ongoing project, I have confirmed that the induction of REDD1 expression in non‐contracted muscle is also observed in mice over 30 weeks old (Figure S3 (https://figshare.com/s/7910d6fd80e7261298f4)), indicating that the findings of this study are not limited to young growing mice but are a universal response to muscle contraction.

Although REDD1 expression is regulated by hormones, including glucocorticoids (Wang et al., 2003, 2006), which are rhythmically secreted in the body, it is unlikely that the increased REDD1 expression in non‐contracted muscles at the time point of 3 h after the contraction is due to its diurnal rhythm. This is because the REDD1 protein in both gastrocnemius and triceps brachii muscles was not increased in non‐contracted control mice euthanized at the same time point as contracted mice (Figure 4). The increase in REDD1 protein was not observed in non‐skeletal muscle tissues, including liver, kidney, and heart (Figure S2 (https://figshare.com/s/7910d6fd80e7261298f4)). These results indicate that isometric contraction transiently induces REDD1 expression in a non‐contracted muscle‐specific manner.

**FIGURE 7:**
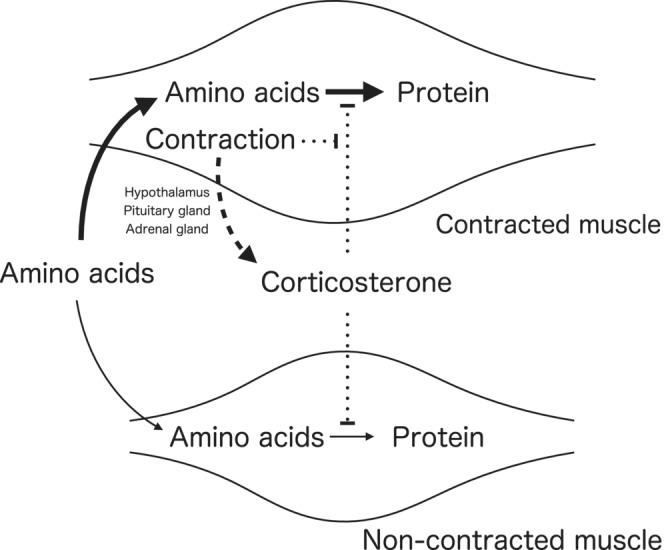
Summary of the present experiment.

The increase in REDD1 protein in non‐contracted muscle suggests the involvement of systemic factors such as the nervous system, hormones, and/or myokines. The skeletal muscle‐specific induction of REDD1 provides a novel insight into the factors and signaling mechanisms that induce REDD1 expression. Glucocorticoids are a potent stimulator of REDD1 expression in muscle (Wang et al., 2003, 2006). In the present study, RU‐486 attenuated the contraction‐induced increase in REDD1 protein and mRNA in non‐contracted gastrocnemius muscle. These results suggest that the hypothalamus‐pituitary–adrenal axis is involved in the contraction‐induced systemic effect to increase REDD1 in non‐contracted muscles (Figure 7).

Previous research has shown that REDD1 protein is decreased 4 h after eccentric contraction (Gordon et al., 2014) in association with enhanced signaling through mTORC1 in muscle. Indeed, the REDD1 protein was decreased 3 h after the contraction in the present study. Gordon et al (2014). showed that the basal expression of REDD1 protein in non‐contracted muscle was higher in fasted animals than fed animals and the contraction decreased the protein to the same extent (~40%–50%) in both conditions. In the present study, I also showed that the contraction decreased REDD1 protein under fed conditions. These results indicate that the contraction indeed decreases the REDD1 protein regardless of whether the basal REDD1 protein is induced by muscle contraction or fasting. Although it has been reported that ubiquitin‐mediated degradation is a mechanism to decrease REDD1 protein (Katiyar et al., 2009), I have shown that contraction leads to a decrease in REDD1 mRNA (Figures 3c and 5b), suggesting that the contraction‐induced reduction in REDD1 protein is regulated at the transcriptional level. Further study is required to elucidate the mechanism by which muscle contraction decreases REDD1 expression in contracted muscle.

Given the role of REDD1 in inhibiting the Akt/mTORC1 pathway (Britto et al., 2020; Manning & Toker, 2017), a transient increase in REDD1 protein in non‐contracted muscles could induce anabolic resistance for protein synthesis in the muscle shortly after the contraction by blunting mTORC1. The temporary decrease in the utilization of amino acids in non‐contracted muscles could increase the availability of amino acids in the contracted muscle for a period of time after the contraction, allowing for macromolecule synthesis, including proteins. This provides insights into the timing of nutrient intake for muscle adaptation to resistance exercise/training, as protein intake after the early cessation of exercise promotes muscle protein synthesis (Levenhagen et al., 2001) more effectively.

In conclusion, isometric contraction transiently increases REDD1 expression in non‐contracted muscles partly through glucocorticoids (Figure 7). The findings suggest a potential mechanism for the involvement of systemic factors, such as the hypothalamus‐pituitary–adrenal axis, in the regulation of REDD1 expression. The results also highlight the skeletal muscle‐specific induction of REDD1 and provide novel insights into the factors and signaling mechanisms that regulate REDD1 expression. Further studies are needed to elucidate the mechanism by which muscle contraction decreases the REDD1 expression in contracted muscles and to explore the potential therapeutic implications of targeting REDD1 in muscle hypertrophy and metabolic disorders.

## FUNDING INFORMATION

This work was supported by JSPS KAKENHI Grant Number JP22K11620.

## CONFLICT OF INTEREST STATEMENT

The author has no conflict of interest concerning this research.

## ETHICS STATEMENT

All animal experiments (protocol 95) were approved by the Experimental Animal Care Committee of Shigakkan University.

## Supporting information


Data S1.

Data S2.
