# Supplementary material for: Isometric contraction induces transient increase of REDD1 expression in non‐contracted muscles partly through glucocorticoids
Source: Physiol Rep. 2023 Jun 6;11(11):e15745. doi: 10.14814/phy2.15745 (PMC10244466; doi:10.14814/phy2.15745)
Supplement: Supplementary file 1 — Data S1. [file PHY2-11-e15745-s002.pdf]

Supplemental Materials and Methods

Free puromycin concentration assay

The concentration of free puromycin was measured as previously described by Goodman et al. (FASEB J. 25: 1028–1039, 2011). A 250-μl aliquot of sample homogenate prepared for Western Blot analysis was precipitated with 28 μl of 100% trichloroacetic acid and incubated on ice for 30 minutes. The mixture was then centrifuged for 5 minutes at 4200g. The resulting supernatant was neutralized by adding 30 μl of 5.25 M NaOH and 15 μl of buffer containing 1 M Tris, 3 M NaCl, and 1% Tween 20 (pH 7.0). The pH of the sample was adjusted to 9.0 with pH test paper. A range of standards (0.025–40.0 pmol in 100 μl) was also prepared by adding free puromycin to the supernatant from samples taken from animals that were not given puromycin injections but were treated exactly as described above. A 100-μl aliquot of the sample or standard was added per well to a 96-well amine-binding maleic anhydride-activated plate (#15100, Pierce; Thermo Fisher Scientific) and rocked overnight at 4°C. The plate was washed four times for 1 minute each with PBS containing 1% Tween 20 (PBST) and then blocked with 1% BSA-PBST for 1 hour at room temperature. Next, 100 μl of anti-puromycin antibody (clone 12D10, #MABE343, Merck Millipore; diluted 1:38,400) in 1% BSA-PBST was added to each well and rocked for 2 hours at room temperature. After four 1-minute washes with PBST, 100 μl of horseradish peroxidase-conjugated anti-mouse IgG in 1% BSA-PBST (#1706515, Biorad; diluted 1:10,000), which was the same antibody used for the SUnSET method, was added to each well and rocked for 1 hour at room temperature. The wells were washed four times for 1 minute each with PBST, and finally, a 100-μl mixture of Biofx Super Sensitive HRP substrate (#TMBS-0100-01, Surmodics) was added. After 15 minutes, the reaction was stopped by adding 100 μl of Biofx 450 nm Stop Buffer (#STPR-100-0, Surmodics) when the blue color developed enough to read. The free puromycin content was calculated and normalized to the protein content within the original 250-μl aliquot of sample using the values from the standard curve.

Supplemental Figure legends

Supplemental Fig. S1. Concentration of free puromycin is not different between contracted and contralateral non-contracted gastrocnemius muscles after isometric contraction at time point 0 h. In the control experiment, a subset of animals were unilaterally contracted as described in “Materials and Methods” in the main text. The incorporation of puromycin into newly synthesized peptides was measured using the SUnSET method. Using four samples (surrounded by color circle) out of the seven samples of the control experiment, the concentration of free puromycin in the muscle was measured by ELISA as described by Goodman et al. Representative immunoblots and the concentration of free puromycin are shown. The sample surrounded by a color circle on the immunoblots corresponds to that on the bar graph. Each connected circle in the bar graph represents data obtained from the same animal. Values are means ± SD. N, non-contracted muscle; IC, isometrically contracted muscle.

Supplemental Fig. S2. REDD1 proteins in liver, kidney, and heart are not different between non-contracted and isometric contracted mice after the isometric contraction at time point 3 h. Representative immunoblots of gastrocnemius muscle lysates were used as a positive control. Black bar between the bands of Immunoblots indicates that membranes are discontinuous. N, non-contracted muscle; IC, isometrically contracted muscle; Contracted, contracted mice, Non-cont, non-contracted mice.

Supplemental Fig. S3. REDD1 protein is increased in non-contracted muscle by isometric contraction in older mature mice. The gastrocnemius muscle of 32 weeks old mice (n = 3) was unilaterally isometric contracted, and the REED1 protein was measured using the same procedure as that for10 weeks old mice, as described in the main text. The increase in REDD1 protein was observed in the non-contracted muscle of older mature mice in response to isometric contraction, similar to that observed in young growing mice. N, non-contracted muscle; IC, isometrically contracted muscle.
