# Supplementary material for: Isometric contraction induces transient increase of REDD1 expression in non‐contracted muscles partly through glucocorticoids
Source: Physiol Rep. 2023 Jun 6;11(11):e15745. doi: 10.14814/phy2.15745 (PMC10244466; doi:10.14814/phy2.15745)
Supplement: Supplementary file 2 — Data S2. [file PHY2-11-e15745-s001.pdf]

Supplemental Table S1. Summary of data of Western blots.

	Time after the contraction (h)										P value of 2 way ANOVA		
	0		3		6		12		24		Main effect		Interaction
	Non-cont	IC	Non-cont	IC	Non-cont	IC	Non-cont	IC	Non-cont	IC	Time	IC	Time X IC
Puromycin incorporation	1.00 ± 0.69	0.18 ± 0.15	1.35 ± 0.63	0.78 ± 0.20	1.14 ± 0.39	1.54 0.72	1.32 ± 0.63	1.35 ± 0.75	0.99 ± 0.93	1.12 ± 1.04	0.3805	0.0556	0.0007
Phospho 4E-BP1 (T37/T46)	1.00 ± 0.24	0.62 ± 0.25	1.08 ± 0.27	1.17 ± 0.18	1.12 ± 0.20	1.17 ± 0.32	1.12 ± 0.17	1.13 ± 0.12	1.12 ± 0.11	1.23 ± 0.13	0.0125	0.4538	0.0009
Phospho S6K1 (T389)	1.00 ± 0.51	1.18 ± 0.39	1.43 ± 0.50	7.93 ± 1.17	1.10 ± 0.49	4.05 ± 1.29	1.33 ± 0.82	4.38 ± 1.53	1.25 ± 0.59	4.86 ± 1.85	< 0.0001	< 0.0001	< 0.0001
REDD1 protein	1.00 ± 0.20	1.02 ± 0.37	7.26 ± 1.26	1.49 ± 0.35	1.79 ± 0.60	1.06 ± 0.24	1.31 ± 0.35	1.15 ± 0.41	1.25 ± 0.57	1.16 ± 0.54	< 0.0001	< 0.0001	< 0.0001

Values are means ± SD for 6 mice in each time point. Values are expressed as relative value to that of Non-contracted muscle at time point 0 h. Non-cont, non-contracted muscle; IC: isometric contracted muscle.

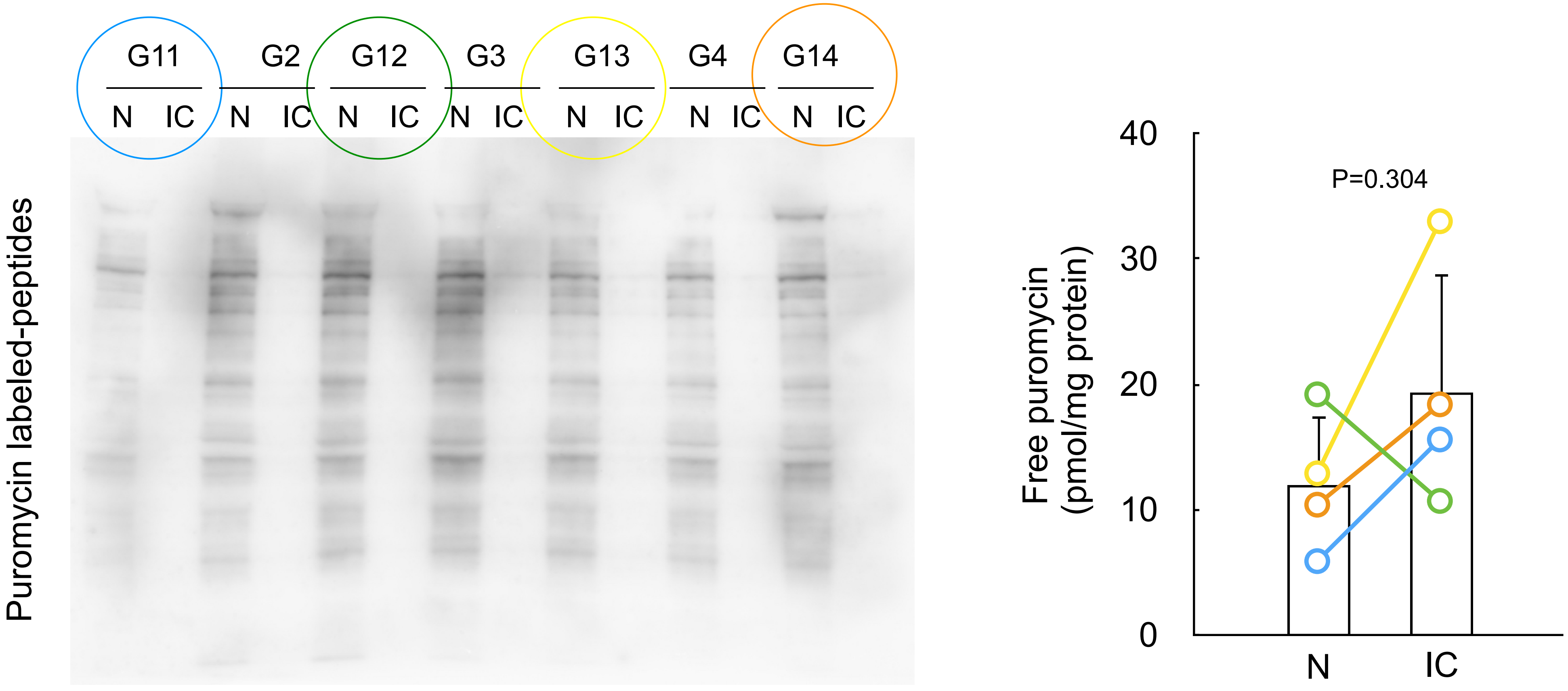

Supplemental Fig. S1. Murakami

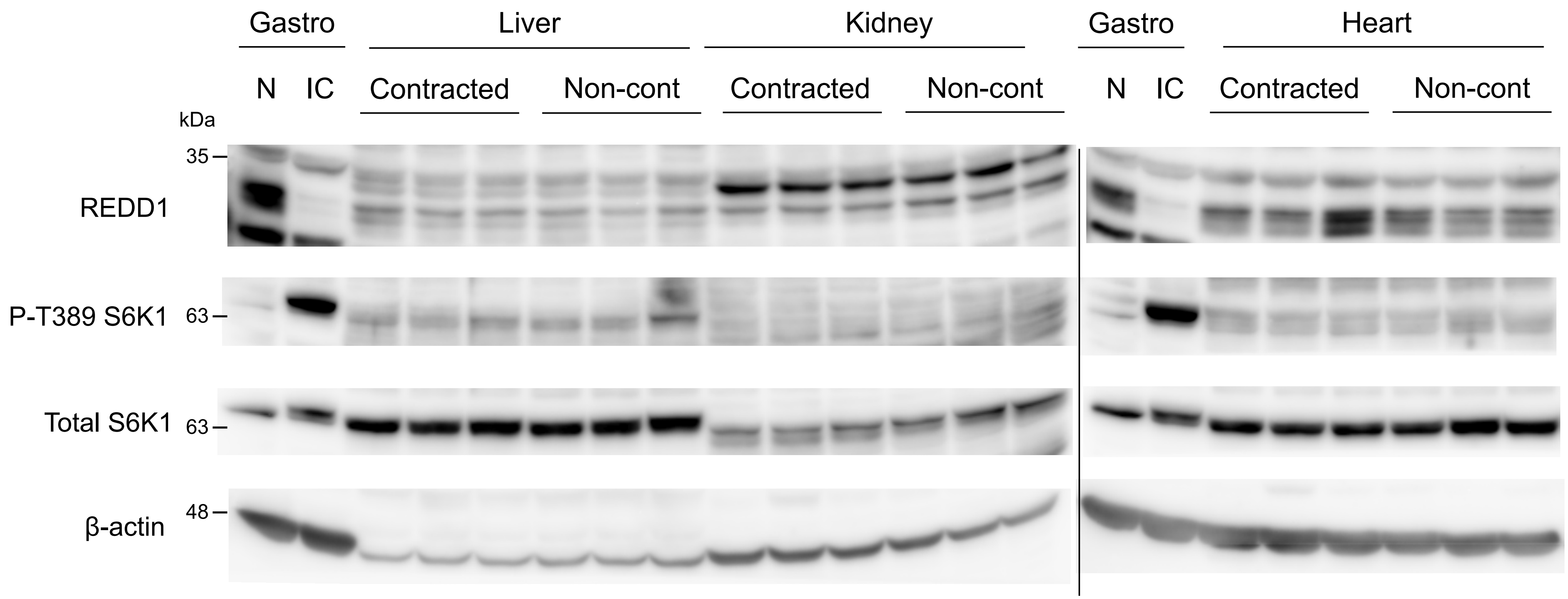

Supplemental Fig. S2. Murakami

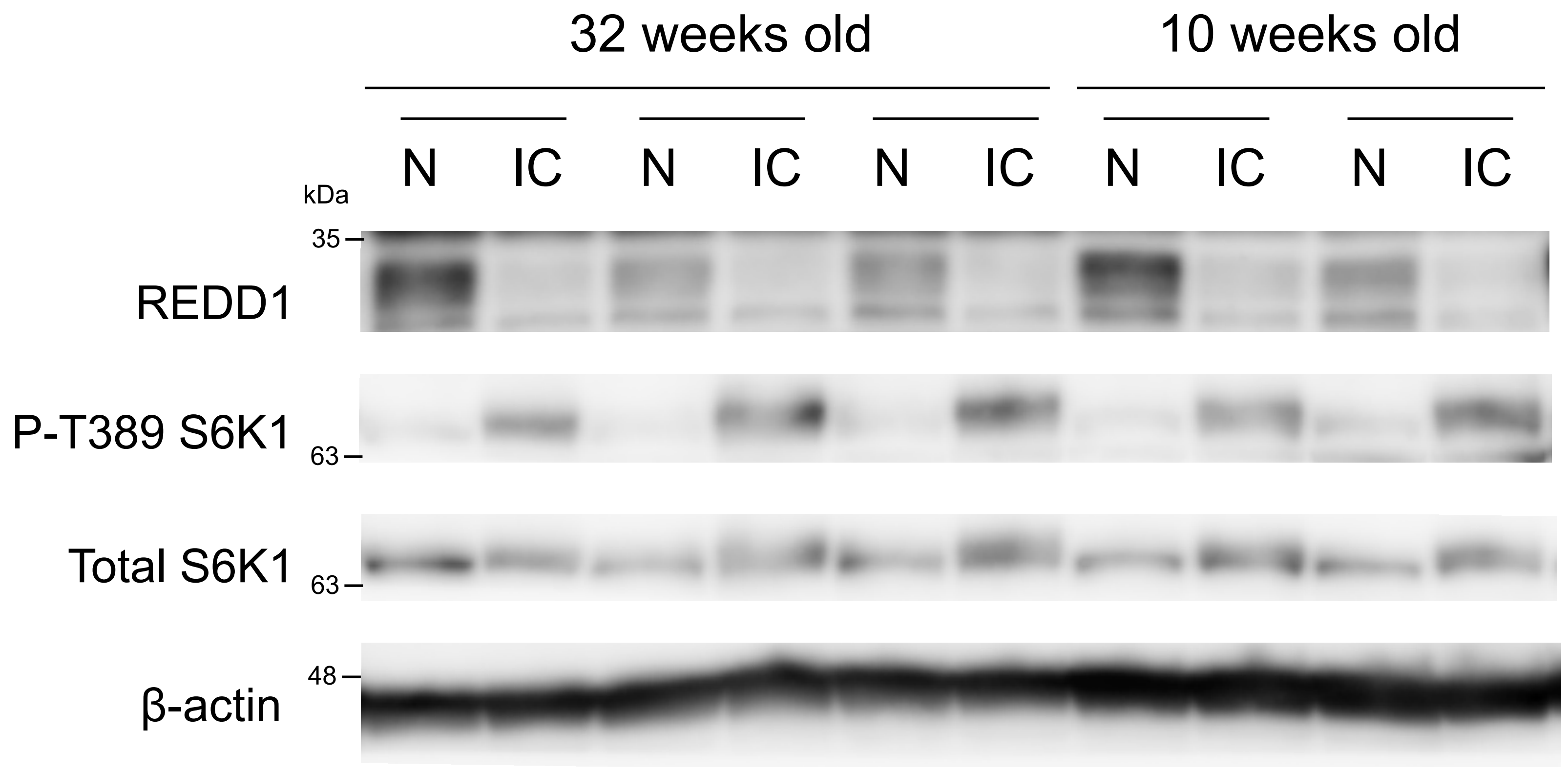

Supplemental Fig. S3. Murakami
